# A Qualitative Exploratory Study of Informal Carers’ Experiences of Identifying and Managing Oral Pain and Discomfort in Community-Dwelling Older People Living with Dementia

**DOI:** 10.3390/geriatrics3030032

**Published:** 2018-06-21

**Authors:** Paul Newton, Charlotte Curl, Ria Prasad, Patricia Pass, Julie Bowden

**Affiliations:** 1 Department of Adult Nursing and Paramedic Science, Faculty of Education and Health, University of Greenwich, Avery Hill Campus, Southwood Site, London SE9 2UG, UK; p.pass@greenwich.ac.uk (P.P.); j.e.bowden@greenwich.ac.uk (J.B.); 2 Special Care Dentistry, King’s College Hospital NHS Foundation Trust, King’s College Dental Hospital, Denmark Hill, London SE5 9RS, UK; charlotte.curl@nhs.net (C.C.); ria.prasad@nhs.net (R.P.)

**Keywords:** informal carers, oral pain and discomfort, identification, management, dementia, older people

## Abstract

Increased prevalence of dementia and poor oral health in older people is associated with more people living with dementia who experience oral pain and discomfort. However, little is known about how informal carers manage oral pain for people living with dementia in the community. This study aimed to explore informal carers’ experiences of identifying and managing oral pain and discomfort in people living with dementia, and barriers and enablers they encountered. Focus groups with informal carers of people living with dementia were conducted, transcribed verbatim, and analysed using thematic analysis. Carers’ accounts suggested that day-to-day contact was required to identify oral pain and discomfort, and a symptomology of the signs and symptoms was developed. Carers’ accounts also highlighted issues in maintaining oral health, difficulties in accessing the mouth, managing dentures, competing demands, and difficulties in accessing treatment due to health service-, behavioural- and treatment- related barriers. Enablers included informal carers’ pivotal role in the identifying and managing oral pain and discomfort in people living with dementia. The study concludes that carers want more partnership work with dental professionals, and clearer care pathways are required to meet the oral health needs of people living with dementia who experience oral pain.

## 1. Introduction

In the United Kingdom (UK) dementia is a growing health concern particularly within the ageing population [1,2]. In 2015, approximately 850,000 people in the UK were estimated to be living with dementia and this is predicted to increase to over 1 million by 2025 and to over 2 million by 2051 [1]. The Adult Dental Health Survey (2009) has revealed that around half of older people (aged over 60) are maintaining some, if not all, of their natural dentition [3]. Although it is a positive health development that people are ‘keeping their teeth’ in old age, there is an increase in the prevalence of tooth decay, gum disease and oral pain and discomfort in the older population—particularly those aged 80 and over [3]. The decline in oral health status, associated with old age—coupled with other factors such as polypharmacy, complex restorative work in situ in the mouth and difficulties maintaining oral health—all contribute to the likelihood of some people living with dementia experiencing oral pain and discomfort. Recent systematic review evidence [4] has found that older people living with dementia have worse oral health, in terms of more retained roots and coronal and root caries, when compared to older people without dementia. Similarly, further systematic review [5] has found that older people with dementia have higher plaque and present a greater level of oral health problems relating to oral soft tissues, e.g., gingival bleeding, periodontal pockets, stomatitis, mucosal lesions, and reduced saliva flow. Few data exist on the prevalence of oral pain and discomfort in people living with dementia in the UK, although some commentators surmise that their oral health may be no poorer than in the wider population, when adjusted for confounding variables [6,7]. Indeed, a recent systematic review [4] found the prevalence of orofacial pain to be equivalent for those living with and without dementia—although the authors note that this may be influenced by to a scarcity of high quality studies exploring orofacial pain and dementia. Due, however, to the communication issues that can arise, oral pain and discomfort are frequently undetected in people living with dementia [6,8]. An early United States (US) study [8], for example, found that up to 60% of a sample of people living with dementia had a dental condition which was likely to cause pain and/or discomfort, and this raises concern about how this may contribute to challenging behaviour issues in this group [4].

In the UK, around two-thirds of people living with dementia are in community ‘own home’ settings [9] and for every 1000 people with dementia in the community there are around 850 people who act as informal carers—caring for spouses, partners, relatives and friends in the community dwelling, i.e., ‘in own home’ [10,11]. Literature exists on training formal carers—i.e., those working in residential homes, or paid to care for a person in their own home—who work with people living with dementia to promote good oral health [10]. Less in known about approaches to ‘informal carers’. One United States study [12] has found that those living at home with dementia are less likely to use dental services, but less is known about why. Equally, many studies comment on the need to: involve and educate all types of carers in oral health, improve pathways to care and develop targeted preventative measures [2,11,12]. Previous studies have also highlighted the need for dental staff to be better prepared when treating patients with dementia [13,14] and to collaborate with other healthcare staff when planning care [15]. Other papers have focused on the clinical identification of orofacial and dental pain, and the use of a suitable diagnostic pain tool [16,17,18]. All three of these studies recommended the development of a pain assessment tool that could help dental professionals identify pain in those who are unable to communicate due to dementia. However, in community dwelling individuals in the UK, day-to-day care is predominantly performed by informal carers. Therefore, the overarching aim of the study was to understand informal carers’ actual experiences of identifying and managing oral pain and discomfort, as well as any barriers and enablers they engendered in seeking help and support. 

As the researchers sought to look at a social process where little is known, and wished to capture informal carers’ day-to-day experiences [19], an exploratory qualitative design was adopted with the further aims of exploring informal carers’ experiences of:

(1) identifying and managing oral pain and discomfort in people living with dementia;
(2) accessing help and treatment for oral pain and discomfort for people living with dementia;
(3) barriers and enablers encountered in identifying and managing oral pain and discomfort in people living with dementia.

## 2. Materials and Methods

A cross-sectional, exploratory qualitative study was conducted, comprised of five focus groups with (N − 6 + 6 + 7 + 8 + 8 = 35) carers of people living with dementia in community dwellings. The study adopted a critical-realist theoretical position, which is premised on the tenet that people have ‘internal conversations’ about how they act [20]. Put simply this means people draw on personal concerns as well as socio-contextual factors when assessing situations and making decisions, i.e., they ‘strike a balance’ between their personal concerns and factors in their social contexts which informs how they act [20]. Focus groups are a method of collecting qualitative data where people are brought together to discuss, or focus on, a specific research topic [21]. It is the interaction between the participants that generates the data (which is recorded), as such the focus group data is analysed to understand how the participants view their social worlds and the meanings they ascribe to events and phenomena [21]. The focus group approach was adopted for two reasons—so that participants could absorb and respond to what each other had said and hence agree the key elements, and through this synergistic process central concerns about the topic could emerge [21]. This approach has been used successfully to explore other primary carers’ experiences of barriers and enablers to managing other health conditions [22] and carers’ experiences of wider dementia care [23].

Qualitative research seeks to purposively find participants that are likely to generate appropriate and useful data, and include enough of them to address the aim of the study [21,24]. Participants were purposively sampled from sites across East Kent in South-East England and all focus groups took place in January to April 2015. Focus group participants were recruited by contacting Dementia Carer Support Groups in Kent and Medway. Of the seven groups approached, five agreed to take part (refusals by groups were for reason of being too busy at the time). In the groups agreeing to take part, the group leader circulated a participant information sheet asking for carers who had experience of looking after somebody living with dementia whilst the person living with dementia had oral pain and discomfort. Where interest was shown, members were told the focus group would be held at one of their upcoming support group meetings and were invited to participate. All participants were in old age, they were predominantly females, with the majority of those being cared for being a spouse, although a few siblings and friends were cared for by those participating. The topic guide for the focus group was developed following a review of the literature to find thematic areas, and following advice from carer organisations and special care dental professionals. The focus groups started with questions on the identification of oral pain and discomfort, e.g., ‘What signs or symptoms did you look for to check whether the person you care for was experiencing dental/oral pain or discomfort?’ The topic guide also moved on to questions relating to managing oral health, and barriers and enablers to seeking help, such as ‘What did you do when you suspected the person you care for had oral pain or discomfort?’ and ‘Where, do you think, would be the best place for treatment to take place if the person you care for was experiencing oral pain or discomfort?’. Finally, questions on the informal carers’ own needs were also covered, e.g., ‘What types of training, if any, do you feel would benefit you, and help you identify or manage oral pain and discomfort in the person you care for?’. Focus groups were conducted by three members of the research team (PN, CC, RP) as well as university staff. All received focus group training. Focus groups lasted around sixty minutes on average.

Data was audio-recorded, transcribed verbatim and analysed inductively for emerging themes, following each focus group—and before the subsequent focus group. Using this process, the focus groups continued until a saturation point was reached—a point whereby the data collected becomes repetitive, so that adding participants is unlikely to generate new ideas [24]. To ensure rigour, transcripts were read by two of the authors (PN and CC) and a framework of emerging themes were developed inductively and in a data-driven way, by negotiating and agreeing on the content, as well as the development of new themes (or subthemes) where there was disagreement. Using this approach, quotes were assigned to themes; hence quotes selected below, represent a given theme. All subjects gave their informed consent for inclusion before they participated in the study. The study was conducted in accordance with the rules of the Declaration of Helsinki, and the protocol was approved by the Ethics Committee of University of Greenwich (Reference No: 16.1.5.19), procedures regarding signed informed consent, anonymity and confidentiality were adhered to throughout the study. 

## 3. Results

Two main themes were identified in relation to carers’ experiences of oral pain and discomfort in people living with dementia:

(1) identification; with two main sub-themes: (A) The need for day-to-day contact and (B) Spotting signs and symptoms (comprising behavioural and physiological changes);
(2) management; with two main subthemes: (A) Maintaining oral health; and (B) Issues of access.

These are discussed in turn below, noting which focus group the quote comes from (e.g., FG1).

### 3.1. Theme 1: Identification

#### 3.1.1. Need for Day to Day Contact

Carers noted subtle changes in behaviour that they felt may indicate oral pain and/or discomfort which manifested itself in day-to-day behaviour and daily routines:

“Because you’re living with each other day by day, you notice little changes”

*(FG1)*

“I’d take him to the doctor but he said it was all fine, but he couldn’t see because it happened at night and after he’d eaten”

*(FG5)*

This suggested that spotting signs and symptoms happened through close day-to day observation by the carers in an everyday context, e.g., noticing pain after eating or when brushing.

#### 3.1.2. Spotting Signs and Symptoms

Carers were asked to describe how they knew when someone living with dementia had oral pain and discomfort, and eight different signs/symptoms were reported which were broadly both behavioural and physiological in nature:

(1) Changes in eating with loss of weight:

Many carers observed changes in weight (physiology) and the eating habits (behaviour) of people living with dementia who were later treated for oral pain and discomfort:

“He couldn’t really eat properly…”

*(FG3)*

“They were off their food … The weight was falling off her …”

*(FG4)*

This lack of eating and related weight loss was clearly due to pain in the mouth preventing eating, which led to weight loss.

(2) Pointing/holding face and facial expressions:

Carers also described frequent facial wincing and cupping of the face as a sign of oral pain and discomfort.

“He suddenly went ‘Ewwww!—like that [participant cups face and squeezes eyes shut]—and he was off-colour, y’know what I mean?”

*(FG1)*

“He would be wincing, covering his face and turning away”

*(FG5)*

These behavioural changes and ‘changes in eating/weight’ were the most commonly reported signs and symptoms, and often led to help-seeking. Carers reported these signs as the clearest signs *to successfully alert* healthcare professionals that the issue may relate to oral pain and discomfort.

(3) Trouble sleeping/relaxing:

Many participants described how people living with dementia were restless at night, pacing until exhausted.

“My husband was up and down all night, couldn’t keep still […] then he was exhausted and out like a light—but when he was sleeping he kept kicking and crying out.”

*(FG2)*

Participants went on to describe that this behaviour abated once treatment was received.

(4) ‘Bad breath’:

The carers were frequently in close proximity to the person living with dementia they cared for and noted that bad breath usually signalled oral health issues.

“I thought there might be an abscess or something in there. I’ve got a very strong nose, and he had bad breath”

*(FG3)*

This was reported as a potential physiological change signalling oral pain and discomfort.

(5) ‘Drooling’:

Another physiological change described was persistent drooling and sucking.

“She’d be sitting in her chair watching telly and you could hear her sucking. There would be a damp patch down her front …”

*(FG2)*

(6) Anger:

“I guess anger is one way my father expressed himself when he was in pain. He’d get very, very agitated and you’d suddenly see him sort of strike out you would think, ‘okay, right!’”

*(FG3)*

However, many carers noted that there was no way to tell if this was related to the oral pain and discomfort.

(7) ‘Body language’ (“jumpy”):

Many participants described how the person they cared for often had a diffuse sense of pain, as if the pain resonated through the person living with dementia’s body and made them sensitive to touch.

“I mean, you just tap him like that [a poking gesture] and he’ll wince, and pull away”

*(FG2)*

(8) Problems wearing dentures:

Another behavioural change reported along with poor eating, jerky body language, bouts of anger, as well as trouble sleeping and relaxing, was problems wearing dentures.

“He hadn’t shaved that morning because he didn’t want to put his dentures in […] I figured it out and dragged him to the dentist but it wasn’t a lot of use.”

*(FG2)*

Here the carer notices a very subtle change in routine which she realises relates to difficulties in putting dentures in her husband’s mouth. This example reinforces the notion that close, one-to-one contact is needed to spot the signs and symptoms.

These signs and symptoms were spotted intuitively by carers and later confirmed by referral to treatment. However, a few carers in the focus groups found it difficult to identify pain in the person living with dementia.

“My husband didn’t seem to recognise pain that well … [I]t’s almost as if that part of his brain had died”

*(FG3)*

Hence, some questioned whether people living with dementia experience pain in the same way as the wider population (particularly in the later stages of dementia).

### 3.2. Theme 2: Management

Carers’ accounts of the management of oral health had two main subthemes: (A) issues with maintaining oral health; and (B) issues of access.

#### 3.2.1. Issues with Maintaining Oral Health

The main problems with maintaining oral health were problems with accessing the mouth, managing dentures, and competing demands on the carers’ time:

(1) Issues with accessing the mouth:

Carers often found accessing the mouth difficult, particularly in later stages of dementia. Carers experienced refusals, and also felt that it was a risk to put their hand in the mouth:

“It they say no and keep their mouth shut, what can you do?”

*(FG5)*

“I’ve been bitten on the wrist before.”

*(FG4)*

In brief, carers reported experiencing poor cooperation and anticipatory risks as barriers to caring for the mouth. Carers themselves acknowledged that they required training not just in technical skills e.g., toothbrushing—but also in behaviour management techniques:

“I wish I could trick him, or you know, coax him, into opening his mouth and letting me get the brush around his teeth. You know, calmly, without all the effort and struggling […] That’s what we need”

*(FG2)*

Hence, behaviour-related barriers may become more problematic as the dementia progressed, but where time and care is taken, carers suggested that these barriers could be overcome.

(2) Issues with managing denture care

Denture management was frequently seen as time-consuming, frustrating and unpleasant:

“They’re pushing you away. Then once you’ve got them out, you’re trying to coax her to get them back in … [if she does it herself] … she would put them upside down—top to the bottom. It used to take two to three days to get them back properly. It is a very awkward subject.”

*(FG1)*

“It’s wet and globby and … unpleasant really”

*(FG3)*

Dentures were also sometimes seen to present a risk:

“She [person living with dementia] likes to clean her dentures but wear them through the night. You see her sleeping with her mouth open and they’re hanging there …”

*(FG4)*

Hence, dentures were seen as both a burden and a hazard. Equally, carers found denture management unappealing. 

(3) Competing demands on time

Carers reported being busy and often had multiple care tasks to manage. As a result, some carers often let oral health slide as a priority:

“… It would take me up to half an hour, chasing around with the brush. And he’d turn his head saying ‘No’ ‘No’ … Then you’ve got to make sure they’re calm before bed or there’s 100 other things piling up. It just drifts”

*(FG2)*

But many carers also realised that good oral health maintenance may make life easier over time, and help them in the future by preventing oral health issues:

“Of course, if their teeth go rotten, they’ll be in pain … and it will affect their diet and you’ll be liquidising everything to make sure they’re getting the goodness”

*(FG1)*

Issues with accessing the mouth, managing denture care and competing demands on carers’ time meant that oral health was sometimes deprioritised. There was some acknowledgement however that oral health should be prioritised to avoid future issues and to prevent reoccurrences of oral pain and discomfort.

#### 3.2.2. Issues of Access

There were 3 subthemes identified in accessing dental management these were: (1) service-related barriers and levers to access; (2) behaviour-related barriers and levers; and (3) dental care experience-related barriers and levers. 

(1) Service-related barriers and levers: 

Many carers reported negative experiences of navigating services, and in relation to consistency in approach by health professionals:

“The dentist sent us to the GP, and the GP sends you back to the dentist and he [husband] won’t co-operate because he’s in pain. But it’s obvious that the GP hasn’t got a clue on the dentistry side …”

*(FG3)*

“… pushed from pillar to post …”

*(FG2)*

Cost was also a barrier for carers:

“The money, it’s a lot of money isn’t it for a set of dentures?”

*(FG3)*

Positive experiences of services were described where the communication, intervention, and referral were decisive, timely and transparent:

“Our dentist sat us down and said ‘This [tooth] needs to come out’ but it was at the back, so she would need to go in [Name of Hospital]. He booked her in straight away […] They had to put her under [general anaesthetic] eventually.”

*(FG2)*

Although carers reported negative experiences of navigating services and concerns about costs as barriers; where services were managed to be receptive and transparent, this ameliorated concerns and provided a facilitator to accessing services.

(2) Behaviour-related barriers/levers

Although many participants reported that people they cared for could self-manage oral care; it was seen as ‘stage-specific’:

“He was good at first then it just fell by the wayside as he got worse …”

*(FG4)*

Behaviour-related barriers were exacerbated with the stage of the dementia, as carers (and in treatment settings—healthcare professionals) were less able to access the mouth. Some carers reported that in dental settings, where dentists performed some simple behaviour management, communication and rapport techniques, these were seen to have positive effects:

“We’ve been to the same dentist about 30 years and they said that a hygienist would see him, and she’s obviously had dementia training because she’s so kind and gentle and does everything really slowly, and he thinks it’s a pleasure going along to see her. So it can work.”

*(FG2)*

Hence, one need identified by carers was low-level behavioural management techniques for both carers and dental professionals.

(3) Dental care experience related barriers/facilitators

A key concern raised by carers was with dentists’ training and skills in dealing with dementia patients:

“There is a total lack of understanding how to treat somebody with dementia, and not looking at the person and the problem.”

*(FG1)*

Other participants reported that the dental environment itself created anxiety for people living with dementia:

“He heard the drill in the waiting room and that was it–he reared up and wouldn’t stay.”

*(FG4)*

Equally, carers wished to be taken seriously by healthcare professionals and to receive acknowledgement for, and support with, managing information, experiences, and expectations for people living with dementia in dental care pathways:

“They [dentist/s] need me there. If they’d realised that, and if they knew about his dementia when I got there, then they wouldn’t keep us waiting for an hour”

*(FG1)*

Conversely, where carers’ concerns and wishes were supported during appointments, treatment sessions were reported as running smoothly.

“They let me stay in with her [during appointment], I hold her hand and we all have a chat. It keeps her calm and she knows she’s safe”

*(FG4)*

On the whole, carers believed dental services should work closely with them, and bring information and services closer to dementia networks:

“Basically they need to be part of the networking system don’t they?”

*(FG1)*

The table below (Table 1) gives an overview of the identification and management issues identified by the study by theme and sub-themes.

## 4. Discussion

The research has contributed new findings to the literature. In terms of identification, the signs and symptoms that the carers felt would indicate oral pain—which were largely based on observable physiological and behavioural indicators that did not require clinical expertise—were accurate and similar to indicators used by healthcare professionals. These emerged from the carers’ day-to day care concerns. Carers’ accounts also described how, and why, they were often best placed to see the signs and symptoms of oral pain and discomfort as they cared for the person all day. As such, carers are potentially invaluable sources of referral and information for healthcare professionals. In terms of maintaining good oral health and preventing oral health issues, carers reported having issues with accessing the mouth, and finding time for oral health. Carers suggested that training in simple behavioural management techniques (alongside existing health education resources focussed on brushing teeth and denture management) would aid them to prevent poor, and maintain good, oral health.

However, the carers also reported socio-contextual barriers and enablers that shaped their ability to manage oral pain and discomfort. The issues emerging in the management theme support previous findings that carers experience barriers to accessing care and treatment [2,9]. This study, however, found that carers’ accounts describe these barriers as: (1) issues with navigating and securing services, and (2) the need for support and training—for both themselves and health professionals—in managing behaviour in existing dental encounters and settings. Carers’ descriptions of enablers and facilitators to accessing oral healthcare, focussed on developing a dementia-friendly, behaviour-management nexus which could be shared by both carers and dental professionals. Hence, whereas previous research has described the need to involve carers in the oral care pathway [2,11,13]—this study has made a first step in identifying how carers feel they can, and should, be involved in this pathway. Participants also felt that they should be valued partners in identification, management and treatment processes. This suggests that the emphasis in the literature that: (1) carers should be involved in designing care pathways [2] and (2) the need for an oral pain assessment tool for people living with dementia [16,17,18] is not only well-founded, but that these two areas could also be mutually informing. Carers are well placed to spot signs and symptoms (especially if aided by an assessment tool), and then want care pathways that are easy to understand and navigate, once they have identified the oral health issue. As noted the signs described by carers were largely based on observable physiological and behavioural indicators that did not require clinical expertise. This should, of course, run in parallel with dementia training for dental professionals which could promote methods of working in partnership with informal carers.

As with all research, limitations exist which may shape the conclusions we are able to draw from the data. First, like all qualitative research, the findings may not be generalisable to the wider informal dementia carer population. Developing a survey instrument to gauge the wider national prevalence of the findings—as well as exploring how the characteristics and social circumstances of informal carers may shape the issues raised by this study—would be fruitful areas of future research. Secondly, special care dentists who were involved in the collection of data made their professional role clear. This could have shaped how participants discussed their experiences of accessing dental care within the focus groups. However, the theme of accessing dental care also emerged frequently, and in the same context and in focus groups conducted without dental professionals present, suggesting their presence was not a confounding issue. Researchers were also encouraged to be reflexive and look at how their social characteristics (e.g., class and professional background) may impact on their relationship to participants and attempt to minimise the ramifications of these. Finally, the participants were carers of people living with dementia in a variety of stages of dementia, who actively engaged in dementia support group services (and already had experiences of help-seeking), carers outside of dementia services and/or looking after people in different stages of dementia may be harder to reach, and thus face different barriers and facilitators to oral healthcare pathways.

## 5. Conclusions

Carers perceived that introducing proactive dementia-friendly procedures within the wider dental treatment environment, and developing training for healthcare professionals aimed at understanding the issues that people living with dementia and their carers experience, were required. Carers also argued that dental professionals should reach out to community-based dementia carer groups to promote good oral health and prevent oral pain and discomfort in people living with dementia. Improving dental care pathways to meet the needs of people living with dementia who experience oral pain is an area requiring urgent further research and development. Equally, having an easy-to-use assessment tool for oral pain and discomfort for people living with dementia could improve detection by informal carers and dental professionals, as well as having potential to facilitate better communication between informal carers, healthcare and dental professionals in clinical encounters.

## Figures and Tables

**Table 1:** Areas of results with themes and subthemes.

Area of Oral Health	Theme	Sub-Themes
**Identifying oral health issues**	A. Need for day-to-day contact	Able to establish deviation from the individual’s ‘norm’
	B. Spotting signs and symptoms	Changes in eating/weight; Pointing/holding face/facial expression; Trouble sleeping/relaxing; ‘Bad breath’; ‘Drooling’: Anger ‘Body language’ (jumpy)/non-verbal/crying: Problems wearing/using dentures
**Managing oral health issues**	A. Maintaining Oral Health	Accessing the mouth: *Refusal and risks* Managing dentures: *Repulsion and risks* Competing demands: *Pressured carers vs preventative care*
	B. Issues of access	Health service-related: *Navigation, Cost & Continuity* Behaviour-related: *Behaviour management/communication* Dental care experience-related: *Dementia friendly environments, Dentists’ networking with Dementia Groups; Improved dementia skills for dentists*
